# Editorial: The gut-liver axis: the main role of microbiome in liver diseases

**DOI:** 10.3389/fmicb.2025.1567513

**Published:** 2025-03-11

**Authors:** Giovanni Tarantino, Tiziana Di Renzo, Mauro Cataldi

**Affiliations:** ^1^Department of Clinical Medicine and Surgery, “Federico II” University Medical School of Naples, Naples, Italy; ^2^Institute of Food Sciences, National Research Council (CNR), Avellino, Italy; ^3^Section of Pharmacology, Department of Neuroscience, Reproductive Sciences and Dentistry, Federico II University of Naples, Naples, Italy

**Keywords:** gut-liver axis, microbiome & dysbiosis, intestinal permeability, liver disease, NAFLD

It is clear that changes in the composition of gut microbiota may contribute to the pathogenesis of liver diseases in multiple ways by influencing gut permeability, immune response, and bile salt metabolism, among other factors (Albillos et al., 2020). Therefore, this Research Topic has been initiated to offer a comprehensive overview of recent advancements regarding the role of microbiota in the multifaceted field of liver diseases, encompassing various conditions such as liver cirrhosis, autoimmune cholangitis, and non-alcoholic fatty liver disease (NAFLD).

Several articles in this Research Topic investigated the relevance of changes in microbiota to the pathogenesis of NAFLD by using completely different experimental strategies. Lin et al. used an animal model of the disease and compared the intestinal microbiome of nine mice with NAFLD induced by a high-fat diet to that of control mice. They found that specific bacteria including *Ileibacterium, Ruminococcaceae, Olsenella, Duncaniella, Paramuribaculum, Bifidobacterium*, and *Coriobacteriaceae_UCG_002* were specifically associated with NAFLD. Different metabolic profiles also corresponded to varying compositions of the gut microbiome. Ultimately, the authors identified a set of 8 bacterial strains, 14 genes, and 83 metabolites that strongly distinguished normal mice from those with NAFLD. In contrast, Pan et al. employed Mendelian randomization with published human data to assess the causal relationship between specific bacterial strains in the gut and the development of NAFLD. The results of this analysis showed that eight bacterial strains are strongly associated with this disease: the bacteria *Actinomycetales*, NB1n, the family *Actinomycetaceae*, the bacteria *Oxalobacteraceae*, and the genus *Ruminococcaceae UCG005* were positively correlated, whereas the family *Lactobacillaceae*, the *Christensenellaceae R7 group*, and the genus *Intestinibacter* were negatively correlated. Additionally, the analysis conducted by Pan et al. revealed that differences in the microbiome were associated with metabolic variations. The results were further validated in a mice model of NAFLD. A markedly different approach was employed by Shera et al., who transplanted fecal matter from patients who had undergone bariatric surgery into mice on a high-fat diet. This treatment significantly reduced the development of NAFLD in these mice and weakened the changes in the cytokine and T-lymphocyte patterns typically associated with this disease.

NAFLD may progress to NASH (non-alcoholic steatohepatitis), which is characterized by significant liver inflammation, and can lead to cirrhosis. The reason this progression occurs in some patients, but not in others, is largely unknown; however, the data reported by Huang et al. in this Research Topic suggest that microbiota composition is a key factor. They discovered that *Megamonas* and *Fusobacterium* were significantly more abundant in the fecal microbiome of NASH patients compared to control subjects and NAFLD patients. The Kyoto Encyclopedia of Genes and Genomes (KEGG) pathway analysis revealed major metabolic differences in the gut microbiota between NAFLD and NASH patients.

The relevance of microbiota changes in the pathogenesis of liver cirrhosis was investigated by Li et al. by comparing microbiome and mycobiome in 45 cirrhotic patients and 30 healthy controls. Distinctive changes were identified; in cirrhotic patients, *Streptococcus, Akkermansia, Ligilactobacillus*, and *Pseudescherichia* were increased, while *Blautia, Anaerobutyricum, Gemmiger, Ruminococcus*, and *Dorea* were decreased. The significant changes in *mycobiota* included *an increase in Saccharomyces* alongside a concurrent decrease *in Aspergillus, Penicillium, Auricularia*, and *Cladosporium*. By combining metagenomic data with the results of metabolomic analyses, the authors developed a diagnostic testing system that was able to identify cirrhotic patients with a high accuracy (area under the curve [AUC] of 0.938 at the receiver operating characteristic [ROC] curve analysis).

In the articles by Cui et al. and Zhang et al., the association between changes in the gut microbiome and autoimmune liver diseases was assessed using Mendelian randomization (specifically for primary biliary cholangitis and primary sclerosing cholangitis).

Natural products have been claimed to exert beneficial effects on liver diseases, which can depend at least in part on changes in the composition of gut microbiota. Data supporting this hypothesis have been reported in two articles of this Research Topic. Sun S. et al. reviewed the evidence that certain traditional Chinese medicine remedies, which are claimed to be effective in treating liver cirrhosis, might be functioning not only as antioxidant, anti-inflammatory, and anti-fibrotic agents but also through modifications in gut microbiota. This has been demonstrated for purified natural products used in traditional Chinese medicine, including polyphenols such as resveratrol, alkaloids such as berberine, terpenoids such as Ginkgo Biloba, carbohydrates such as alginate, and glycosides such as FTA, a glycoside extracted from the dried fruit of *Forsythia suspensa*. Evidence of beneficial changes in microbiota composition is also available for herbal prescriptions that contain multiple compounds from various sources. Liang et al. investigated the impact of licorice on the hepatoxicity of Evodiae Fructus—a medicinal herb with analgesic, anti-tumor, and anti-inflammatory properties. They demonstrated that, in mice, Evodiae Fructus, particularly in the small-flowered forms, induces oxidative damage and inflammation in the liver. Additionally, these effects are reduced when the extract is administered along with licorice extracts. Changes in microbiota composition may contribute to licorice protective action. Licorice prevented the decrease in the abundance of *Corynebacterium* induced by Evodiae Fructus, while simultaneously causing an increase in *Candidatus Arthromitus*. Importantly, the abundance of these two bacterial genera correlated positively and negatively with biochemical markers of oxidative damage and inflammation, respectively. These results not only emphasize that natural products may protect the liver by changing gut microbiota but also highlight that toxic substances may harm the liver in a microbiota-dependent way. This concept was also addressed in the article by Tarantino et al., which explores the role of gut microbiota in the hepatotoxicity induced by drugs of abuse. The authors reviewed the literature demonstrating that opioids change the composition of intestinal microbiota by increasing Gram-positive bacterial species, including *Staphylococcus sciuri, Staphylococcus cohnii*, and *Staphylococcus aureus*, as well as *Enterococcus durans, Enterococcus casseliflavus, Enterococcus faecium*, and *Enterococcus faecalis*. This opioid-induced dysbiosis leads to the weakening of the intestinal barrier and, as a result, allows bacterial species to translocate to the liver. This occurrence strongly promotes inflammation and cell damage. Importantly, data are available indicating that similar mechanisms also contribute to alcohol-induced hepatotoxicity. If the hypothesis that gut dysbiosis plays a significant role in the pathogenesis of alcohol-induced liver damage is correct, then it could be suggested that interventions aimed at modifying the composition of intestinal microbiota may be beneficial for this condition. Wei et al. explored how alcohol feeding induces gut dysbiosis in mice by increasing the abundance of *Oscillibacter, Escherichia/Shigella, and Alistipes*. This increase is accompanied by a parallel rise in liver T-helper (Th) 1 and Th17 lymphocytes, along with a decrease in Regulatory T-cells (Treg) cells. These changes were completely reverted by the combined administration of *Akkermansia muciniphila* and inosine, a compound that acts as an energy source and also provides anti-inflammatory and immunomodulatory effects. Remarkably, this combined treatment promoted the growth of butyrate-producing bacteria, including not only *Akkermansia* but also *Lactobacillus* and *Clostridium IV*.

**Figure 1:**
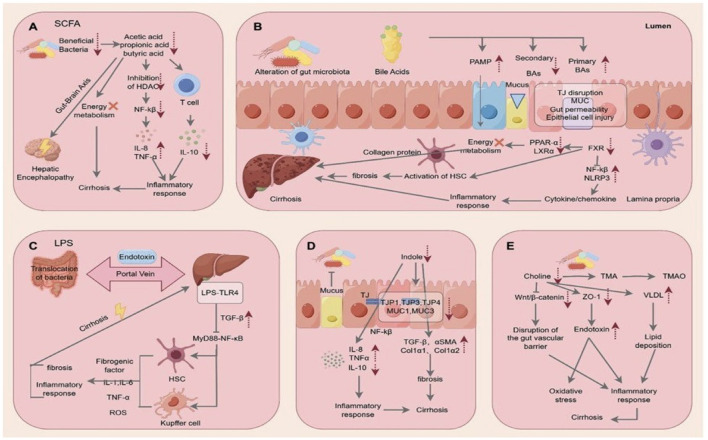
Multiple mechanisms by which the liver–gut axis may exert protective or detrimental effects on the liver: **(A)** Short-chain fatty acids; **(B)** Bile salts; **(C)** Endotoxin and bacterial translocation; **(D)** Indole; **(E)** Choline and trimethylamine (TMA) (Reproduced from the article by Sun S. et al. published in this Research Topic).

The molecular mechanisms by which intestinal microorganisms may exert beneficial or detrimental effects on the liver have been extensively investigated and crucial factors have been identified, such as bacterial translocation and endotoxin release, the release of indole and trimethylamine, bacterial metabolism of biliary acids, and changes in the level of short-chain fatty acids (Figure 1). The article by Sun R. et al. describes a new potential mechanism for liver protection involving the gut microbiome. By using quadrupole time-of-flight mass spectrometry (Q-TOF-MS)-based metabolomics, they demonstrated that, in mice, liver regeneration following partial hepatectomy is associated with a significant increase in liver steroid hormone biosynthesis, potentially linked to the observed rise in *Escherichia, Shigella, Lactobacillus, Akkermansia*, and *Muribaculaceae* within the gut microbiome.

In this Research Topic, we aimed to improve our understanding of the potential mechanisms that explain the connection between gut flora dysbiosis and liver damage. The 16 publications collected in this Research Topic have expanded our understanding of the microbiome's role and its involvement in liver diseases. Further exploration is needed to determine if modifying the composition of gut microbiota with probiotics, prebiotics, or symbiotics could ameliorate or prevent liver diseases.
